# Characterization of K_6_Ta_10.8_O_30_ Microrods with Tetragonal Tungsten Bronze-Like Structure Obtained from Tailings from the Penouta Sn-Ta-Nb Deposit

**DOI:** 10.3390/nano10112289

**Published:** 2020-11-19

**Authors:** Belén Sotillo, Lorena Alcaraz, Félix A. López, Paloma Fernández

**Affiliations:** 1 Department of Materials Physics, Faculty of Physics, Complutense University of Madrid, 28040 Madrid, Spain; arana@ucm.es; 2 National Center for Metallurgical Research (CENIM), Spanish National Research Council (CSIC) Avda. Gregorio del Amo 8, 28040 Madrid, Spain; alcaraz@cenim.csic.es (L.A.); f.lopez@csic.es (F.A.L.)

**Keywords:** mining tailings, tantalum, X-ray diffraction, Raman spectroscopy, scanning electron microscopy

## Abstract

In this work, a deep characterization of the properties of K_6_Ta_10.8_O_30_ microrods has been performed. The starting material used to grow the microrods has been recovered from mining tailings coming from the Penouta Sn-Ta-Nb deposit, located in the north of Spain. The recovered material has been submitted to a thermal treatment to grow the microrods. Then, they have been characterized by scanning electron microscopy, X-ray diffraction, micro-Raman and micro-photoluminescence. The results of our study confirm that the K_6_Ta_10.8_O_30_ microrods have a tetragonal tungsten bronze-like crystal structure, which can be useful for ion-batteries and photocatalysis.

## 1. Introduction

Tantalum is a highly appreciated material in several technological applications. Following the studies performed by European Union (EU) about this element [1], it is typically used in superalloys for propulsion systems, due to the high strength and resistance to corrosion, or in the electronics industry. Tantalum oxide system is important for electronic applications. While tantalum has metallic character, tantalum oxide is a dielectric material with a high dielectric constant. The combination of metal and dielectric is the basis of the tantalum capacitors [2].

A problem faced by the industry that make use of tantalum-based materials is that tantalum is an element scarcely found in the Earth’s crust. In the case of the EU, mines where the raw material can be extracted (from columbotantalite, for instance) are a rarity. On the other hand, the main producer countries have unstable political situations [3], making it difficult for industry to access this material and causing market price volatility. For these reasons, EU considers tantalum as one of the critical raw materials for the economy [1].

The Penouta Mine (Galicia, Spain), exploited by Strategic Minerals Spain S.L. to obtain tin has recently been unveiled as a promising source for other minerals, in particular coulombotantalite [4]. Our team is working in the recovery of tantalum and niobium from the mining tailings, in order to reintroduce these precious raw materials into the technological life cycle [5].

**Figure 1:**
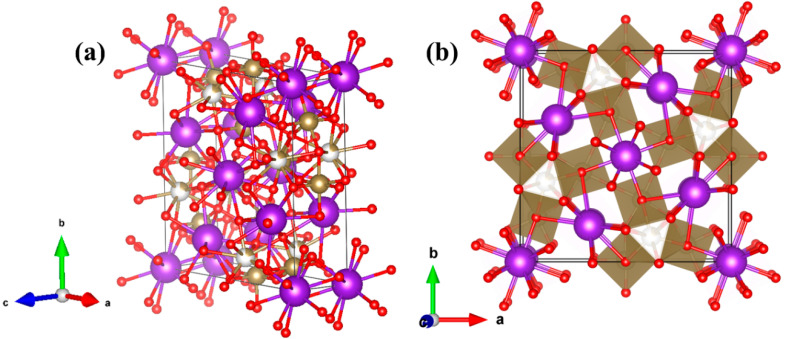
(**a**) K_6_Ta_10.8_O_30_ tetragonal structure simulated plotted using VESTA software [16]. Red, purple and gold balls represent oxygen, potassium and tantalum atoms respectively (**b**) K_6_Ta_10.8_O_30_ structure oriented along the c-axis.

During the process to recover the tantalum metal from the tailings, an intermediate product, potassium-poor tantalate (K_6_Ta_10.8_O_30_) with interesting properties is also obtained. This material has been rarely studied in the literature [6,7,8,9]. We have studied the properties of this material and used it for the growth of microrods. Our interest in this material is mainly related to its peculiar crystal structure (Figure 1a). It has a partially “filled” tetragonal tungsten bronze-like structure, as described by Awadalla et al. [7]. This structure is characterized by having crystallographic channels or tunnels along the c-axis (Figure 1b). Although the tunnels are occupied by K ions in this structure, it is still possible to use them for the insertion of Li ions without producing a large alteration of the crystal structure [10]. This makes K_6_Ta_10.8_O_30_ structure a good anode candidate for Li-ion or Na-ion batteries [10,11,12,13,14] and other electrochemical energy storage devices [15] or photocatalysts [8].

## 2. Materials and Methods

**Figure 2:**
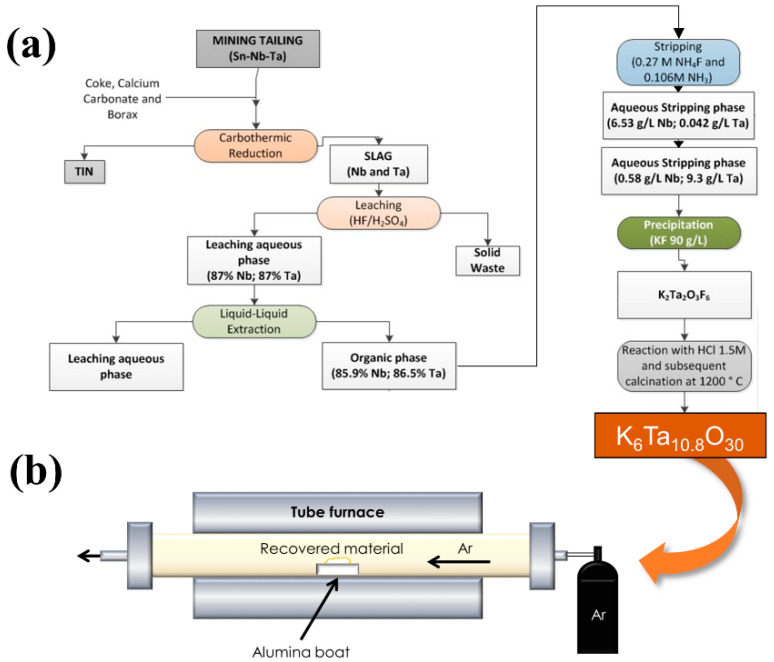
Scheme of the synthesis of potassium tantalate (K_6_Ta_10.8_O_30_) recovered material (**a**), and the subsequent thermal treatment to obtain the microrods (**b**).

The recovery of the Ta-compounds from the tailings have been previously reported [5]. As a summary of the process, Penouta mining tailings (cassiterite and columbotantalite) were treated by a pyrometallurgical process to obtain a metal tin ingot and a slag [4]. The slag was subsequently treated by acid leaching. This was carried out using 6 M solutions of HF (Hydrofluoric acid 40% for analysis, ISO; PanReac AppliChem) and H_2_SO_4_ (Sulfuric acid 95–98% pure, pharma grade; PanReac AppliChem). One hundred mL of 6 M HF and 170 mL of H_2_SO_4_ per litre of leach solution were used. The slag/leaching agent ratio was 100 g/L. This acid leaching gives rise to a liquid aqueous phase with an approximate Ta and Nb content of 87% and a solid residue. This solution may be considered the starting point for Nb and Ta oxides. In this paper we will focus on Ta compounds, however, the first steps of the synthesis routes are common for both oxides, as schematized in Figure 2. The extraction of oxides from the leaching aqueous phase were performed by liquid-liquid extraction using 35% (*v*/*v*) Cyanex 923^®^ (Cyanex 923 Extractant; Solvay, Brussels, Belgium) diluted in Solvesso. Then, the use of two different stripping solutions leads to the separation of both of Nb- and Ta-compounds. After performing the first stripping of Nb (stripping solution with NH_4_F 0.3 mol/L and NH_3_ 0.1 mol/L), the stripping solution used had a composition of NH_4_F 1.1 mol/L and NH_3_ 0.4 mol/L. Once the Ta was separated, solid precursors were obtained by precipitation with KF. Solids were treated after precipitation with HCl (Hydrochloric acid 37% technical grade; PanReac AppliChem) 1.5 M and subsequently calcined at 1200 °C, to obtain the final product K_6_Ta_10.8_O_30_. Figure 2a shows schematically the steps of the synthesis process. Further information about the recovery of this material can be found in Rodríguez et al. [5].

In order to obtain the microrods, thermal treatments have been performed in a tubular furnace (Figure 2b). The powders of the recovered material were placed on an alumina boat and introduced in the furnace under a continuous Ar flow of 2 L/min. The treatment duration was set to 8 h, and the temperature varied between 1300 °C and 1500 °C. The temperature was raised to the final value at a rate of 900 °C/h.

The recovered material and the microrods have been characterized by X-ray diffraction (XRD), energy dispersive X-ray microanalysis (EDX), electron backscattered diffraction (EBSD), micro-Raman (µ-Raman) spectroscopy and micro-photoluminescence (µ-PL) to assess their composition and crystal structure. All the measurements were done at room temperature. For performing the analysis on the microrods, they have been deposited on a silicon <100> substrate.

X-ray Diffraction (XRD) measurements have been done by means of an Empyrean diffractometer (PANalytical, Almelo, The Netherlands) in the Bragg-Brentano geometry using Cu-Kα radiation, with a step in 2θ of 0.05°. For EDX measurements, a QUANTAX 70 detector (Bruker, Berlin, Germany) attached to a TM3000 system (Hitachi High Technologies Corporation, Tokyo, Japan) working at 15 kV has been employed. The EDX data have been analyzed using the Bruker ESPRIT QUANTAX EDS software. EBSD measurements have been carried out with a Bruker *e*^−^Flash Detector coupled to an Inspect-S SEM instrument (FEI Company, Eindhoven, Netherlands) working at 20 kV. The analysis of the EBSD data has been performed with the commercial ESPRIT QUANTAX CrystAlign software.

µ-Raman measurements have been carried out in a LABRAM-HR confocal microscope (Horiba JobinYvon, Villeneuve d’Ascq, France) using the 632.8 nm line of a He-Ne laser. The laser was focused onto the sample using a 100× objective (0.9 NA, Olympus, Tokyo, Japan), and the scattered light was collected using the same objective (backscattering configuration). The grating used to analyse the signal had 600 L/mm, and the signal was collected with an air-cooled charge coupled device camera (CCD). µ-Raman spectra were collected and analysed using the Labspec 5.0 software (Horiba, France, Labspec version 5.0). Finally, luminescence properties and optical microcavity behavior of the microrods have been tested using µ-PL measurements. µ-PL has been collected with the same setup as µ-Raman but using the 325 nm line of a He-Cd laser and a 40× UV objective (0.47 NA, Thorlabs, Newton, NJ, USA).

## 3. Results and Discussion

**Figure 3:**
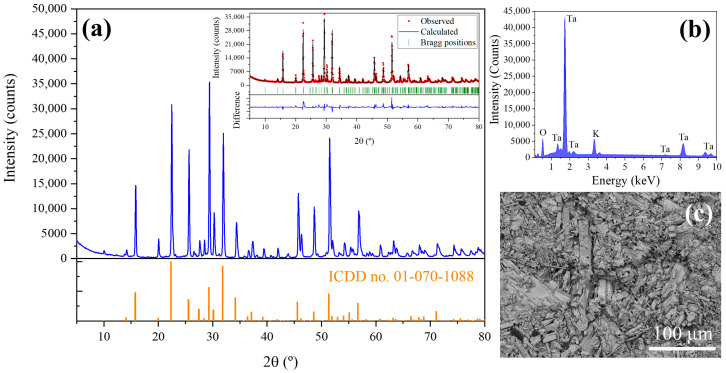
(**a**) XRD pattern of the K_6_Ta_10.8_O_30_ recovered material. The expected position of the peaks, simulated from the ICDD card no. 01-070-1088, are presented below with orange bars. In the inset, observed (red circles) and calculated (solid black line) XRD patterns after Rietveld fitting in the tetragonal space group P4/mbm, with computed lattice parameters *a* = *b* = 12.58 Å and *c* = 3.96 Å. The vertical green marks give the positions of the allowed Bragg reflections, the difference between the calculated and measured patterns is shown below (blue line). (**b**) EDX spectrum of the recovered material, confirming the presence of tantalum (Ta), oxygen (O) and potassium (K). (**c**) SEM image of the K_6_Ta_10.8_O_30_ recovered material.

X-ray diffraction has been performed to determine the crystalline phases present in the precursors. XRD pattern is shown in Figure 3a. The peaks observed in XRD diagrams have been adjusted by Rietveld analysis (inset in Figure 3a) to the peaks of K_6_Ta_10.8_O_30_ tetragonal tungsten bronze-like structure (ICDD card no. 01-070-1088, space group P4/mbm) [7]. The computed lattice parameters are shown in Table 1. Also, a preferential orientation in (410) planes is observed (peak at 29.4°). EDX analyses (Figure 3b) performed on the powders show the expected peaks corresponding to Ta, K and O (11.9 at% of K, 23.9 at% of Ta and 64.2 at% of O) as corresponds to the stoichiometry of the phase observed in the XRD experiments.

Figure 3c shows a SEM image of the material obtained after precipitation and calcination (last steps in Figure 2a before the thermal treatment in the furnace). In this image it can be seen that the recovered material have crystalline particles with a high dispersion in sizes and shapes, and the most identifiable shapes are rods of irregular habits.

**Figure 4:**
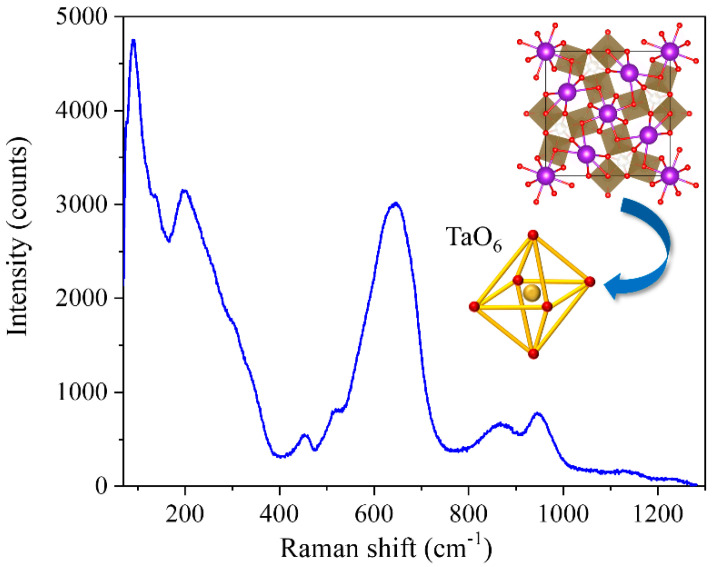
Typical µ-Raman spectrum recorded on the recovered K_6_Ta_10.8_O_30_ material. The wavelength of the probe is 632.8 nm, and the objective used to excite the sample and to collect the signal is a 100×.

The μ-Raman spectra recorded on the recovered material are shown in Figure 4. To the best of our knowledge, there are no previous studies where the Raman spectrum of K_6_Ta_10.8_O_30_ is described. In order to identify the vibration modes in the spectra, we have made a comparison with the Raman characteristics of other compounds with similar crystal structure. To perform this, we need first to describe the crystal structure [7] of the material (Figure 1). The skeleton of the structure can be seen as a weave of corner-linked TaO_6_ octahedra (see inset of Figure 4). The spatial distribution of the octahedra leaves different types of tunnels (with trigonal, square or pentagonal shapes) where other metal atoms can be positioned. More Ta atoms are located at the trigonal-shape tunnels, whereas K ions fill the pentagonal and square tunnels, forming the structure represented in Figure 1.

In comparison with previous literature of crystal structures with similar units [17,18,19,20,21,22], we can divide the Raman spectrum into four different frequency regions: low ($\nu <100\;{\mathrm{cm}}^{-1}$); medium-low ($100\;{\mathrm{cm}}^{-1}<\nu <450\;{\mathrm{cm}}^{-1}$); medium-high ($450\;{\mathrm{cm}}^{-1}<\nu <900\;{\mathrm{cm}}^{-1}$); and high ($\nu >900\;{\mathrm{cm}}^{-1}$). In the $\nu <100\;{\mathrm{cm}}^{-1}$ region, the modes associated with the vibrations of the K-O bonds are found, since K ions are loosely bounded to the oxygen atoms surrounding the tunnel and the long bond distance [7,23]. In the rest of the frequency regions, most of the observed bands can be related to the internal modes of TaO_6_ polyhedra. Typically, the external lattice vibrations (for which the octahedra is considered as a rigid unit) are found at $\nu <150\;{\mathrm{cm}}^{-1}$ [17,18]. In the medium-low and medium-high frequencies, the bending and stretching vibrations of the TaO_6_ polyhedra are found. As the stretching modes usually appear at higher frequencies than the bending modes, the $100\;{\mathrm{cm}}^{-1}<\nu <450{\;\mathrm{cm}}^{-1}$ would correspond to the bending related modes of the TaO_6_ octahedra, Ta-O-Ta bending modes appearing at the lower frequencies of this range, while at the higher frequencies the O-Ta-O bending modes would be found [20,23]. It has also been proposed that the band around 200 cm^−1^ could have some contribution from TaO_6_-K bond modes [24].

In the $450\;{\mathrm{cm}}^{-1}<\nu <900\;{\mathrm{cm}}^{-1}$ region we can found the modes related to the stretching vibrations of the Ta-O bonds in the TaO_6_ octahedra. The broad band centered at 645 cm^−1^ is quite common in compounds with octahedral units [21,25], and is normally related to the symmetric stretching mode in the octahedra. The large width of this band comes from the distortion that the octahedra can have in this kind of crystal structures, producing a wide range of frequency vibrations. The band located at 865 cm^−1^ is typically related to antisymmetric stretch of Ta-O-Ta bridging bonds [21] or to breathing vibrations of the TaO_6_ octahedra [24]. This last assignation comes from observations done in perovskite-type compounds, and the possibility to observe this vibration in the Raman spectrum is related to the slight distortion of the octahedra [26]. Finally, the band centered at 945 cm^−1^ (and in general, bands that appear at frequencies above 900 cm^−1^) is ascribed to the symmetric stretching of –Ta=O terminal bonds in the structure [21,22,25].

**Figure 5:**
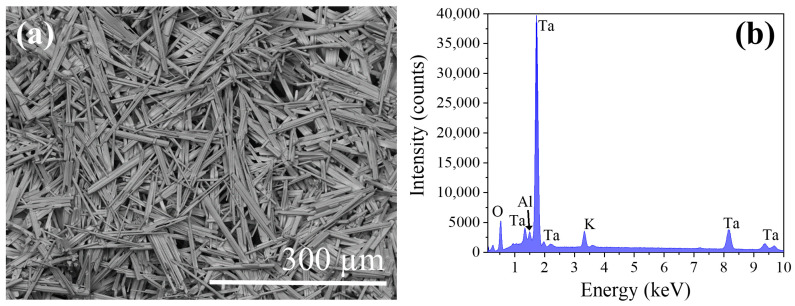
(**a**) SEM images of the K_6_Ta_10.8_O_30_ microrods. (**b**) EDX spectrum recorded on the microrods.

After the determination of the properties of the recovered material, it has been submitted it to a thermal treatment in order to obtain microstructures with more uniform morphologies. As mentioned in the experimental section, thermal treatments have been performed at different temperatures ranging from 1300 °C to 1500 °C. However, the optimum conditions were determined to be 1500 °C, during 8 h under a continuous Ar flux. After the thermal treatment a high density of microrods is obtained (Figure 5a). The size and habit distributions are now much narrower than prior to the thermal treatments. The rods have a rectangular cross-section, with lengths around 150–200 μm and lateral dimensions about 20 μm. As for the starting material, EDX analysis has been performed, showing the same element distribution, with only a weak impurity peak associated to Al (Figure 5b). This impurity comes from the alumina boat where the powders were placed to perform the treatments.

**Figure 6:**
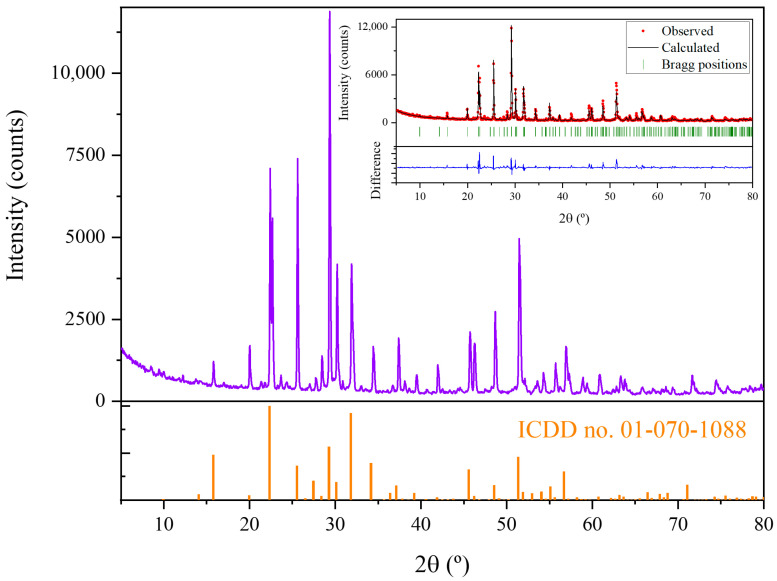
XRD pattern of the K_6_Ta_10.8_O_30_ rods. The expected position of the peaks, simulated from the ICDD card no. 01-070-1088, are presented below with orange bars. In the inset, observed (red circles) and calculated (solid black line) XRD patterns after Rietveld fitting are shown. The vertical green marks give the positions of the allowed Bragg reflections, and the difference between the calculated and measured patterns is shown below (blue line).

XRD performed on the samples after the thermal treatments are shown in Figure 6. Again, the measured peaks can be ascribed to the K_6_Ta_10.8_O_30_ tetragonal tungsten bronze-like structure, confirming that the material has not suffered any crystal phase transformation. The estimated lattice parameters from the Rietveld fitting are presented in Table 1. Slight variations in the value of the lattice parameters may be related to strain introduced during the growth process of the rods. The main difference with the XRD pattern of Figure 3a is the strongest preferential orientation in the (410) reflection, which can be related to the fact that now in the sample there are rods with a well defined crystallographic orientation (Figure 5a).

**Figure 7:**
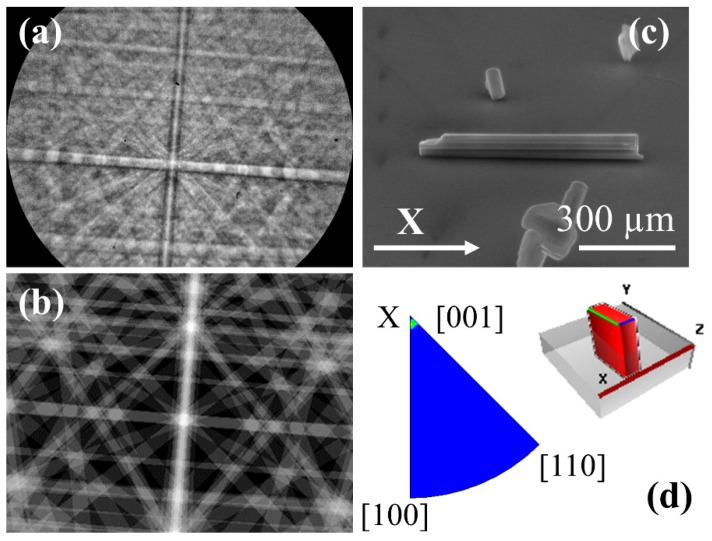
EBSD measurements performed on a K_6_Ta_10.8_O_30_ microrods. Measured (**a**) and simulated (**b**) Kikuchi pattern of a rod, associated to the tetragonal K_6_Ta_10.8_O_30_ phase. (**c**) SEM image of the rod, indicating the X axis of the measurement system. (**d**) Inverse pole figure (IPF) in the X direction, showing that the main crystallographic orientation along X is [001], which is the growth direction of the rod. The inset shows the orientation of the tetragonal cell, associated to the rod, respect the X, Y, Z axis of the microscope system.

From XRD measurements, we can obtain information on the predominant crystal structure of the rods. However, to confirm the crystal structure of individual structures and to determine the growth directions, EBSD measurements have been also performed (Figure 7). An example of the Kikuchi pattern obtained from the rods is presented in Figure 7a. The pattern can be assigned, using the CrystAlign software, to the K_6_Ta_10.8_O_30_ tetragonal tungsten bronze-like structure. In Figure 7b we show the simulated Kikuchi pattern for the same crystal orientation and experimental conditions as the pattern shown in (a). All the patterns recorded on the same microrod are similar, confirming that, in general, we have single crystal structures. In order to get information about the growth direction of the rods, they have been oriented along the X-axis of the reference system of the microscope, as shown in Figure 7c (the Kikuchi pattern shown in Figure 7a is recorded on this rod). If several Kikuchi patterns are measured on different points along the rod, the inverse pole figures (IPF) can be plotted using again the CrystAlign software. These IPFs show the distribution of the main crystallographic directions that are closer to the X, Y, Z directions of the reference system (i.e., reference system of the microscope). Then, checking the IFP for the X-axis, the crystallographic direction that is closer to that axis can be obtained (Figure 7d). From this IPF, it has been determined that the growth direction is [001], i.e., the *c*-axis direction, as shown in the scheme of Figure 7d. This growth direction has been observed also in K_6_Ta_10.8_O_30_ nanowires [6].

**Figure 8:**
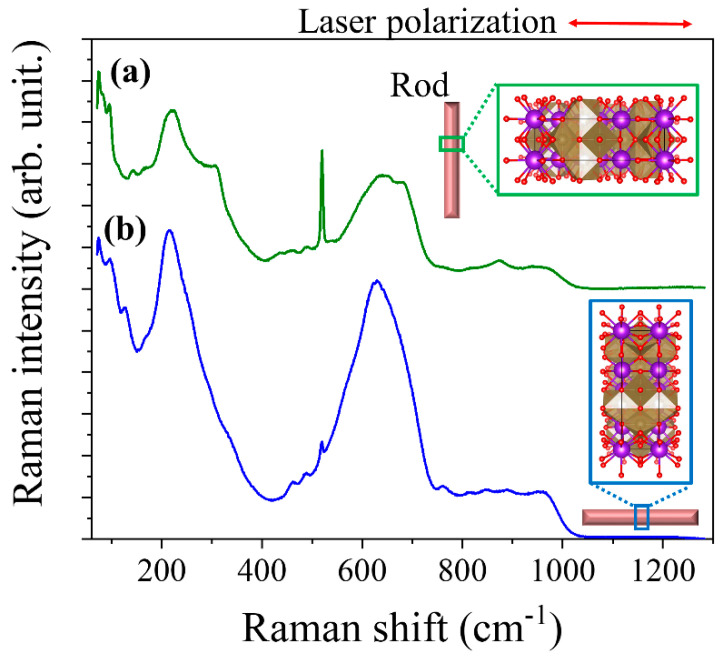
µ-Raman spectra recorded on individual rods, showing the effect of the orientation of the laser polarization respect to the long axis of the rod: (**a**) laser polarization perpendicular to the growth axis of the rod; (**b**) laser polarization parallel to the growth axis of the rod.

μ-Raman spectra have been recorded from individual rods. Figure 8 shows the typical unpolarized Raman spectra obtained. It has been observed that the spectrum varies as the relative orientation of the rod changes respect to the direction of the laser polarization. In other words, when the rod is oriented with the growth axis parallel (perpendicular) to the laser polarization, it means that the polarization is parallel (perpendicular) to the [001] crystallographic direction (see insets in Figure 8). The K_6_Ta_10.8_O_30_ microrods are mostly monocrystalline, as confirmed by EBSD measurements, so the Raman spectra obtained are expected to be polarization sensitive. Spectrum (a) corresponds to the rod oriented parallel to the polarization of the incident laser, while (b) corresponds to the rod oriented perpendicular to the polarization. This effect was not observed in the precursor powders, as those spectra were recorded not from isolated rods but from bundles of particles with different orientations, obtaining a sum of the spectra for all orientations. The sharp peak that appear at 520 cm^−1^ comes from the silicon substrate.

As the composition and crystal structure are the same for both the rods and the precursors, the Raman modes observed in the rods have the same ascriptions as for the precursor (previously described in this work). As have been said before, most of the observed modes are related to the internal vibrations of the TaO_6_ octahedra. So, depending on the polarization configuration and the selection rules, some Raman modes will be visible or not [27]. The possible distortion of the octahedral and the surroundings will also affect the selection rules (see inset in Figure 8). From the spectra shown in Figure 8, it can be seen that vibrations located in medium-low frequencies range ($100\;{\mathrm{cm}}^{-1}<\nu <450\;{\mathrm{cm}}^{-1}$) and the band centered at 645 cm^−1^ are the modes more sensitive to the polarization.

**Figure 9:**
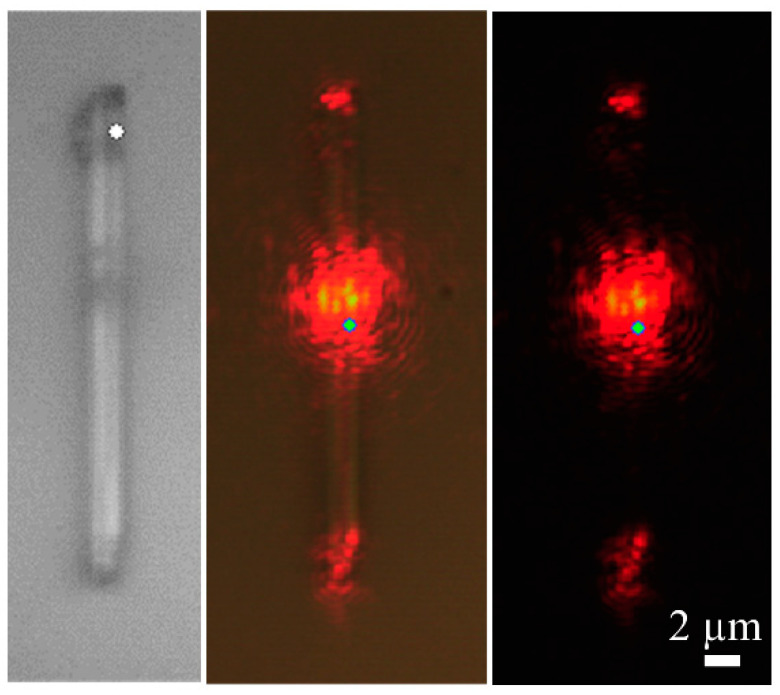
Optical microscope images (100× objective) showing the 633 nm light guiding of a potassium tantalum oxide rod.

Finally, light guiding and confinement properties of the microrods have been studied. The 632.8 nm red laser of the confocal microscope has been used to check the light guiding behaviour of different rods. An example is shown in Figure 9. We have observed that the light is guided from one edge to the other of the rod, or to both edges of the rod if the excitation is source is located at the centre (Figure 9). This indicates that the refractive index of the rods is high enough to confine efficiently the light inside them.

**Figure 10:**
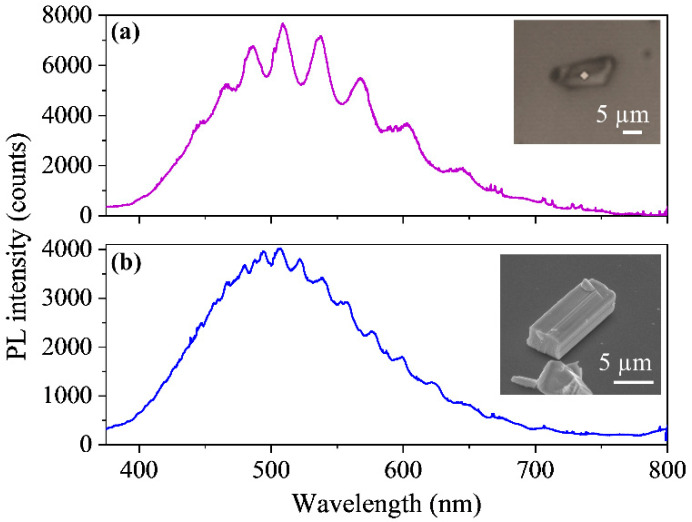
µ-PL spectra (*λ_exc_* = 325 nm) recorded on two microrods with two parallel facets. The confinement of the light between these two facets produces Fabry-Pérot optical resonances, which appear overlapped on the PL emission. (**a**) Rod with distance between parallel facets of 2.7 ± 0.6 µm. (**b**) Rod with distance between parallel facets of 4.6 ± 0.3 µm.

Since the cross sections of the rods are, in general, irregular, it is hard to find structures showing an optical microcavity behaviour as found in other systems [28,29,30]. To perform this experiment, a 325 nm laser is used to locally excite the PL of the rod, using a 40× objective. Thereby the light confined in the optical cavity is the luminescence emission of the material. In Figure 10 the μ-PL spectra recorded on two rods with different dimensions are presented. 

In both cases, the rods have at least two parallel facets, and the light is confined between these two facets (the distance between facets for rod (a) is 2.7 ± 0.6 µm, and for rod (b) is 4.6 ± 0.3 µm). As the light is being reflected between two parallel facets, it is a Fabry-Pèrot optical cavity. The wavelength positions of the optical resonances, which appear in the PL spectra (Figure 10), are determined by the following equation:

(1) $$\lambda =\frac{2hn}{N}$$

Being *h* the distance between the parallel facets (2*h* is the optical path), *n* the refractive index and *N* the interference order. But the refractive index also depends on the wavelength. Previous studies of optical resonances typically start with a tabulated value of refractive index to determine the value of *N*, and then estimate a dispersion relation for the refractive index [28,29]. To the best of our knowledge, there are no previous reports on the refractive index values on K_6_Ta_10.8_O_30_. Then, we are going to perform an approximation of Equation (1), in order to obtain an estimated value of the refractive index of our material. In we consider n constant in a small range of wavelengths, then the distance between two adjacent resonant peaks is:

(2) $$\Delta \lambda \approx \frac{\lambda^{2}}{2hn}$$

Equation (2) shows that the larger is h the smaller is the distance between optical resonances Δλ, in agreement with the measurements presented in Figure 10 for the two rods. Using the peaks that appear at the maximum of the PL emission, which are the narrowest and the most defined (located at 510 ± 1 nm and 537 ± 1 nm for rod (a) and at 506 ± 1 nm and 522 ± 1 nm for rod (b)), we can obtain an estimation of the refractive index:

$$\mathrm{Rod}\;\left(a\right):\;n\approx \frac{\lambda^{2}}{2h\Delta \lambda }\approx 1.9\pm 0.4\;@\;523\;\mathrm{nm}$$

$$\mathrm{Rod}\;\left(b\right):\;n\approx \frac{\lambda^{2}}{2h\Delta \lambda }\approx 1.9\pm 0.3\;@\;514\;\mathrm{nm}$$

The value obtained for refractive index is similar for both rods. To the best of our knowledge, this is the first value reported for the refractive index on K_6_Ta_10.8_O_30_.

## 4. Conclusions

In this work, we have recovered a tantalum compound from mining tailings coming from the Penouta Sn-Ta-Nb deposit in Northern Spain. The analysis of the material has demonstrated that it is K_6_Ta_10.8_O_30_ with tetragonal tungsten bronze-like crystal structure. Using the recovered material, we have obtained microrods by a thermal treatment of the material at 1500 °C in an Ar flux. To the best of our knowledge, it is the first time that this material (K_6_Ta_10.8_O_30_) has been studied by means of EBSD, μ-Raman and μ-PL. From EBSD measurements, the growth direction of the microrods have been determined to be along the *c*-axis direction. The possible origins of the vibration bands observed in Raman spectra have been discussed, being most of the modes related to the vibrations of the TaO_6_ octahedra. Finally, a value of the refractive index of the material in the visible region has been estimated from the μ-PL measurements. Then, we have shown that the microrods can be used as light guides or as optical resonant microcavities. All these results will be useful for the future use of this material in Li-ion or Na-ion batteries or for photocatalysis.

## Figures and Tables

**Table 1:** Estimated lattice parameters for recovered material and for rods.

	*a* (Å)	*b* (Å)	*c* (Å)
Recovered material	12.58	12.58	3.96
Rods	12.59	12.59	3.93
